# *ADAM9* Expression in Uterine Cervical Cancer and Its Associated Factors

**DOI:** 10.31557/APJCP.2019.20.4.1081

**Published:** 2019

**Authors:** Seoparjoo Azmel Mohd Isa, Md Salzihan Md Salleh, Mohd Pazudin Ismail, Suhaily Mohd Hairon

**Affiliations:** 1 *Department of Pathology, *; 3 *Department of Obstetrics and Gynecology,*; 4 *Department of Community Medicine, School of Medical Sciences, *; 2 *Hospital Universiti Sains Malaysia, Universiti Sains Malaysia, Malaysia. *

**Keywords:** ADAM proteins, ADAM9 protein, *ADAM9* expression, uterine cervical carcinoma

## Abstract

**Background:**

Cervical cancer is a preventable disease caused by human papillomaviruses. It is the third most common cancer to occur in women of reproductive age. The ADAM9 protein plays a role in basement membrane degradation and tumour metastasis in certain types of tumour. Thus, it has the potential to become a new targeted therapy. The objective of this study was to investigate *ADAM9* expression in cervical cancer and to determine the factors associated with *ADAM9*-positive expression.

**Methods:**

This cross-sectional study was conducted in Hospital Universiti Sains Malaysia (HUSM) Kelantan, Malaysia from December 2010 to December 2012. Histological slides obtained from 95 cervical cancer cases diagnosed and/or treated in HUSM from 2000 to 2010 were analysed. The ADAM9 immunostain was then performed on the paraffin blocks. The statistical data entry and analysis were done using SPSS version 18.0. Multiple logistic regression analysis was performed to determine the factors associated with *ADAM9*-positive expression.

**Result:**

Of the 95 cervical cancer patients included in the study, 72 (75.8%) patients showed positive *ADAM9* expression. The mean age of the patients was 53.89 (10.83) years old. Squamous cell carcinoma was the most common type of cervical cancer (n = 67, 70.5%). Factors that showed a statistically significant association with *ADAM9*-positive expression were tumour size (adjusted odds ratio [adj. OR]: 1.08; 95% confidence interval [CI]: 1.02, 1.13; p = 0.004), distant metastasis (adj. OR: 12.82; 95% CI: 1.91, 86.13; p = 0.009) and the histological type of cervical cancer (i.e. squamous cell carcinoma) (adj. OR: 7.39; 95% CI: 1.42, 38.51; p = 0.017).

**Conclusion:**

The ADAM9 immunostain was consistently positive in malignant cells. Thus, *ADAM9* expression can be used as a prognostic/therapeutic indicator in aiding clinician decision-making regarding patient treatment (targeted therapy).

## Introduction

Cervical cancer is the fourth most frequent cancer in women worldwide, with an estimated 530,000 new cases in 2012, representing 7.9% of all female cancers. According to the World Health Organization (WHO), 90% of 270,000 cervical cancer-related deaths in 2015 occurred in low- and middle-income countries. The majority of these deaths occurred in women of reproductive age. These findings have implications for both the social and economic fabric of countries worldwide. Although the statistical data reveals a worrying trend in deaths, cervical cancer is preventable and treatable, either by surgery, radiotherapy, chemotherapy or combination therapy (Bruni et al., 2017). In Malaysia, cervical cancer is the third most common cancer among women, and it accounted for 7.7% of cancers in women between 2007 and 2011. The incidence trend increased among women aged 35 years and peaked at the age of 50–74 years (Manan et al., 2015).

The World Health Organization (WHO) divides cervical carcinoma into various subtypes: epithelial tumours, mesenchymal tumours, mixed epithelial and mesenchymal tumours, melanocytic tumours, miscellaneous tumours, lymphoid and hematopoietic tumours and secondary tumours. It further classifies epithelial tumour into a number categories: squamous tumours and precursors, glandular tumours and precursors and other epithelial tumours (Tavassoli and Devilee, 2003).

Squamous cell carcinomas are the most common type of epithelial cancer of the cervix, which accounts for 80% of total cervical cancers, followed by adenocarcinomas (15% of total cervical cancers) and adenosquamous carcinomas. Other types of carcinomas comprise about 5% of all cervical cancers. All the common types of carcinoma of the cervix (i.e. squamous cell carcinomas, adenocarcinomas and adenosqamous carcinomas) are caused by high oncogenic human papilloma viruses (HPVs) (Bruni et al., 2017). 

The most common types of HPVs associated with cervical cancer are HPV-16, -18, -33, -45, -31, -58, -52 and -35. Fifty-four percent of squamous cell carcinomas and 41% of adenocarcinomas of the cervix are associated with HPV-16 infection, whereas 11% of squamous cell carcinomas and 37% of adenocarcinomas of the cervix are associated with HPV-18 infection. HPV-16 and -18 are the most common etiological agents of squamous cell carcinomas and adenocarcinomas of the cervix, respectively. In terms of infection, the HPV invades the basal cell layer with the help of receptors (heparin sulphate proteoglycans and α6-integrin) in dividing basal cells. The viral particles are then integrated with the host (basal cells) DNA during replication. HPV E6 and E7 proteins play an important role in the development of cervical cancer. These proteins inhibit apoptosis and prevent DNA damage repair . Apoptosis of basal cells that express the E6 is inhibited, thereby resulting in an altered response to DNA damage and the accumulation of genomic mutations. In most cases, HPV infection resolves spontaneously. However, in cases of repeated infection and re-infection and unresolved infection, the infection may progress and lead to high grade intra-epithelial lesions (squamous cell carcinomas, adenocarcinomas or adenosquamous carcinomas) and cervical cancer (Gonzalez-Martin, 2007). 

Cell proliferation may lead to disease spread (i.e. metastasis) in various cancers, including cervical cancer. Cell proliferation and disease progression are caused by the mutation and production of various proteins and enzymes (Malur et al., 2001). The protein A disintegrin and metalloproteinase (ADAM) belongs to a newly discovered group of proteins involved in tumour proliferation and progression. These ADAM proteins and matrix metalloproteinases share a similar sequence with that of reprolysin, a snake venomase. The proteins play a role in various biological functions, such as cell adhesion, fusion and migration, in addition to membrane protein shedding and proteolysis. Many ADAM proteins are expressed in malignant tumours, where they are involved in the activities of growth factors and functions of integrin. The activities of these proteins lead to the promotion of tumour cell growth and invasion (Mochizuki and Okada, 2007; Murphy, 2008).

*ADAM9* overexpression is associated with high-grade and aggressive tumours, tumours with distant metastasis, poorly differentiated tumours and tumours that have a poor prognosis. In a study of hepatocellular carcinomas and colon cancer, *ADAM9* expression was increased. High *ADAM9* expression in cancer was associated with a more aggressive phenotype (Mazzocca et al., 2005). ADAM9 was also highly expressed in renal cell carcinomas. It was associated with high-grade tumours and distance metastasis in renal cell carcinomas (Fritzsche et al., 2008). In poorly differentiated adenocarcinomas of the pancreas, the ADAM9 protein was also strongly expressed. In addition, overexpression of the ADAM9 protein was observed in pancreatic ductal adenocarcinoma, with a poor prognosis (Grutzmann et al., 2004). Overexpression of the ADAM9 protein was reported in non-small cell carcinoma of the lungs, which metastasized to the brain (Shintani et al., 2004).

Furthermore, *ADAM9* expression was found in cases of cervical intra-epithelial carcinomas and in the squamous cell carcinomas. The expression of *ADAM9* increased in accordance with cell malignancy malignant (i.e. staining was stronger in squamous cell carcinomas than in normal cervical cells) (Zubel et al., 2009).

Previous research demonstrated that ADAM9 has uses in targeted therapy. In research involving prostate cancer patients, those who had *ADAM9* expression benefited more from targeted therapy (Josson et al., 2011). In recurrent breast cancer patients, *ADAM9* overexpression served as a marker of the response to tamoxifen therapy (Sieuwerts et al., 2005). Cervical cancer cells’ culture is inhibited after treated with miR-126, a micro-RNA that specified to target *ADAM9* gene. Upregulation of this micro-RNA had a negative effect on cervical cancer cell proliferation (Yu et. al, 2013). Therefore, the objective of the present study was to investigate the proportion of *ADAM9* expression in three common types of cervical cancer and to study the factors associated with *ADAM9*-positive expression.

## Materials and Methods

*Study design and sample size*

This cross-sectional study was conducted in Hospital Universiti Sains Malaysia (HUSM), Kelantan, Malaysia between December 2010 and December 2012. The sample size was calculated using a one sample proportion formula, with a type I error of 5%, type II error of 20%, precision of 5.5% and based on the reported prevalence of *ADAM9* positivity of 93% The required sample size was considered 93 patients, given a 10% drop out. 

*Data collection*

Tissues from cervical cancer patients diagnosed and/or treated in HUSM between 2000 and 2010 were used in this study. The patients’ socio-demographic data and disease history were obtained from case notes held in the records office of HUSM. Paraffin blocks were obtained from a collection available in the pathology department. ADAM9 immunostain was performed on the tissue sections that were produced from the paraffin blocks.

The inclusion criteria for specimens were as follows: 1) a diagnosis of cervical cancer and/or treatment for cervical cancer in HUSM between 2000 and 2010 and 2) a specimen classified as epithelial cervical cancer in one of the three main sub-types (i.e. a squamous cell carcinoma, adenocarcinoma or adenosquamous carcinoma). The exclusion criteria were as follows: 1) cases with incomplete socio-demographic data or an incomplete disease history due to missing patient-related data or unavailable laboratory data; 2) cases with no or missing paraffin blocks and 3) cervical cancer types other than squamous cell carcinoma, adenocarcinoma and adenosquamous carcinoma. The specimens were sampled using the convenient sampling method. All available and eligible samples based on the inclusion and exclusion criteria were included in this research.

*ADAM9 immunostaining*

ADAM9 immunostaining of 4 µm thick tissue sections placed on poly-L-lysine glass slides was performed. The tissue sections were cut from the corresponding paraffin block and float mounted on the glass slide. The section on the slide was warmed on a warming plate for 5 min to liquefy the paraffin. It was then deparaffinised using xylene and hydrated in graded ethanol solution to water. Antigen retrieval was performed using the pressure-cooking method according to the protocol specified by the pathology laboratory of Universiti Sains Malaysia (pH 9.0 citrate buffer for 30 min).

Once the antigen retrieval process was complete, the slide was removed from the pressure cooker and cooled to room temperature under running tap water. After the cooling process was complete, the slide was mounted into the wet chamber for subsequent processing. The slide was washed with tris-buffered saline (TBS) (pH7.6). Hydrogen peroxide was applied for 5 min, followed by washing twice with TBS. The ADAM9 antibody (goat polyclonal ADAM9, clone C-15; Santa Cruz Biotechnology Inc) with dilution of 1:50 was applied to the slide. The slide with the ADAM9 antibody was incubated overnight (8 h) at 4O°C. The next day, the slide was washed twice with TBS. Subsequently, secondary antibody (anti-goat antibody; Santa Cruz Biotechnology) with a dilution of 1:200 was applied for 30 min, followed by washing twice with TBS. The slide was then removed from the wet chamber and placed on a rack, with the tissue side facing up. Subsequently, the slide was flooded with the chromogen 3, 3′-diaminobenzidine and left for 5 min at room temperature and then washed under tap water. The slide was then counterstained with hematoxylin. It was dipped for 5 min and placed under running tap water for 5 min. Once the hematoxylin staining was complete, the slide was dehydrated again using graded ethanol and finally xylene. For immunostaining, in accordance with the protocol proposed by Zubel et. al, all brown cytoplasmic cell staining was considered positive, despite the intensity of the stain. Based on this protocol, the stains were classified as positive if a weak, moderate or strong stain were detected and negative otherwise Two pathologist evaluated the immunostained slides independently. Disagreement was resolved by consensus based on an evaluation of the slides under a multi-headed microscope under 40 times magnification.

*Statistical analysis*

The statistical data entry and analysis were done by using SPSS version 18.0. The data are presented as mean and standard deviation (SD) for numerical variables and as the frequency, with the percentage (%) for categorical variables. The data were analyzed using simple and multiple logistic regression analyses. The outcome variable (binary) was *ADAM9* expression: *ADAM9*-negative expression (control) or *ADAM9*-positive expression (case). Simple logistic regression was used to select the preliminary variables associated with *ADAM9*-positive expression. Variables with a p-value of < 0.25 or any clinically relevant were included in the multiple logistic regression analysis. Multiple logistic regressions were used to evaluate the factors associated with *ADAM9*-positive expression. A preliminary main effect model was obtained after comparing the model using backward likelihood ratio and forward likelihood ratio methods. The fitness of the model was tested using the Hosmer and Lemershow goodness of fit test, a classification table and a receiver operating characteristic curve. The significance level was set at 0.05.

## Results

Due to missing samples, the final study consisted of 95 samples of cervical cancer. The majority of the cases were Malays (n = 76, 80.0%) and non-smokers (n = 85, 89.5%), without co-morbid diseases (n = 65, 68.4%), distant metastasis (n = 54, 56.8%) or lymph node involvement (n = 71, 74.7%). A squamous cell carcinoma was the most common type of cervical cancer (n = 67, 70.5%). The mean age of the subjects was 53.89 (10.83) years old. The patients’ profiles are shown in Table 1.

Of the 95 subjects included in this study, there were 72 cases of *ADAM9*-positive expression. The proportion of patients with *ADAM9*-positive expression was 75.8% (95% confidence interval [CI]: 67.2%, 84.4%). In the study, there were 25 (26.3%) cases of adenocarcinoma, 3 (3.2%) cases of adenosquamous cell carcinoma and 67 (70.5%) cases of squamous cell carcinoma.

**Figure 1:**
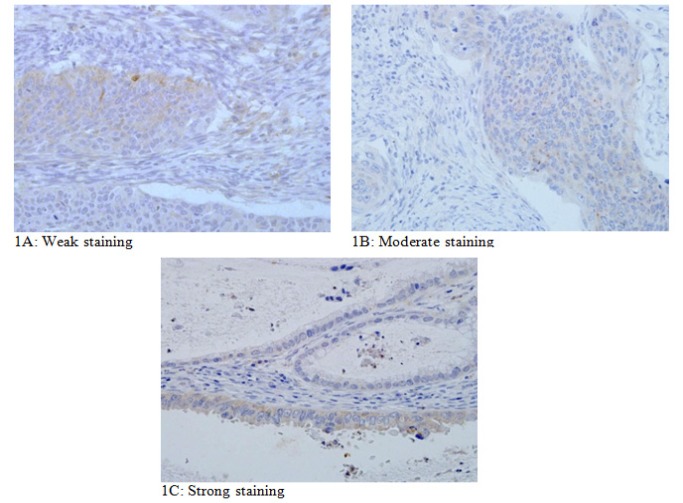
Degree of *ADAM9* Staining Reactivity

**Table 1:** Profile of Cervical Cancer Patients (n=95)

Variables	*ADAM9* negative expression n(%)	*ADAM9* positive expression n(%)	Cervical patients n(%)
Age (years old)	52.22 (9.35)	54.43 (11.28)	53.89 (10.83)*
Ethinicity			
Malay	19 (82.6)	57 (79.2)	76 (80.0)
Chinese	4 (17.4)	11 (15.3)	15 (15.8)
Others	0 (0.0)	1 (1.4)	4 (4.2)
Age of first pregnancy (years old)	22.09 (2.95)	21.81 (2.35)	21.87 (2.49)*
Number of children	5.04 (2.18)	4.56 (2.07)	4.67 (2.10)*
Smoking history			
Non-smoker	21 (91.3)	59 (88.1)	85 (89.5)
Smoker	2 (8.7)	8 (11.9)	10 (10.5)
Co-morbid disease			
Absent	14 (60.9)	51 (70.8)	65 (86.4)
Present	9 (39.1)	21 (29.2)	30 (31.6)
Histological type of cervical cancer			
Adeno CA	7 (30.4)	18 (25.0)	25 (26.3)
Adenosquamous CA	1 (4.3)	2 (2.8)	3 (3.2)
Squamous cell CA	15 (65.2)	52 (72.2)	67 (70.5)
Tumor size (mm)	23.04 (14.02)	40.13 (16.25)	35.99 (17.30)*
Lymph nodes involvement			
No	21 (91.3)	49 (68.1)	71 (74.7)
Yes	2 (8.7)	23 (31.9)	24 (25.3)
Distant metastasis			
No	21 (91.3)	33 (45.8)	54 (56.8)
Yes	2 (8.7)	39 (54.2)	41 (43.2)
ADAM9 expression			
Negative			23 (24.2)
Positive			72 (75.8)

*mean(sd)

**Table 2:** Factor Associated with *ADAM-9 *Positive Expression by Simple Logistic Regression (n=95)

Variables	*ADAM9* negative expression n=23 n(%)	*ADAM9* positive expression n=72 n(%)	Crude OR (95% CI)	p-value
Age (years old)	52.22 (9.35)	54.43 (11.28)	1.02 (0.98, 1.07)	0.393
Ethnicity				
Malay	19 (82.6)	57 (79.2)	1	
Chinese	4 (17.4)	11 (15.3)	0.92 (0.26, 3.22)	0.892
Others	0 (0.0)	4 (5.5)	5384 (0.00, 0.00)	0.999
Smoking history (n=67)				
Non-smoker	21 (91.3)	59 (88.1)	1	
Smoker	2 (8.7)	8 (11.9)	1.31 (0.26, 6.67)	0.743
Co-morbid disease				
Absent	14 (60.9)	51 (70.8)	1	
Present	9 (39.1)	21 (29.2)	0.64 (0.24, 1.71)	0.373
Lymph nodes involvement				
No	21 (91.3)	49 (68.1)	1	
Yes	2 (8.7)	23 (31.9)	10.33 (1.31, 81.37)	0.027
Tumor size (mm)	23.04 (14.02)	40.13 (16.25)	1.08 (1.04, 1.11)	<0.001
Histological type of cervical cancer				
Adeno CA	7 (30.4)	18 (25.0	1	
Adenosquamous CA	1 (4.3)	2 (2.8)	0.78 (0.06, 10.00)	0.847
Squamous cell CA	15 (65.2)	52 (72.2)	1.35 (0.47. 3.83)	0.575
Distant metastasis				
No	21 (91.3)	33 (45.8)	1	
Yes	2 (8.7)	39 (54.2)	12.41 (2.71, 56.89)	0.001

**Table 3:** Factor Associated with *ADAM-9* Positive Expression by Multiple Logistic Regression (n=95)

Variables	*ADAM9 *negative expression	*ADAM9* positive expression	Adjusted OR (95% CI)	p-value
	n=23 n(%)	n=72 n(%)		
Tumor size (mm)	23.04 (14.02)	40.13 (16.25)	1.08 (1.02, 1.13)	0.004
Histological type of cervical cancer				
Adeno CA	7 (30.4)	18 (25.0	1	
Adenosquamous CA	1 (4.3)	2 (2.8)	0.38 (0.01, 19.76)	0.629
Squamous cell CA	15 (65.2)	52 (72.2)	7.39 (1.42, 38.51)	0.017
Distant metastasis				
No	21 (91.3)	33 (45.8)	1	
Yes	2 (8.7)	39 (54.2)	12.82 (1.91, 86.13)	0.009

OR, odds ratio; CI, confidence interval; Adj, adjusted. Backward LR method applied. Hosmer and Lemeshow test, p-value=0.838. classification table 95.8% correctly classified. Area under Receiver Operating Characteristics (ROC) curve was 0.843. No multicollinearity and no interaction found

Figure 1A shows weak staining (a faint brown colour) in clusters of malignant cells. The stain of the stroma was considered negative, as no brown colour was seen. Whereas Figure 1B shows moderate staining, as determined by the intensity of the brown colour. The strongest staining of tumour cells is shown Figure 1C, with marked brown-coloured cytoplasmic cells clearly visible.

The results of the simple and multiple logistic regressions, which were performed to identify the factors associated with *ADAM9*-positive expression, are displayed in Tables 2 and 3. According to the final model obtained after employing the backward selection procedure, the odds of *ADAM9*-positive expression were 7.39 times more likely in patients with squamous cell carcinomas versus those patients with adenocarcinomas (95% CI 1.42, 38.51; p = 0.017). Cervical cancer patients who had distant metastasis had 12.82 times chance of having *ADAM9*-positive expression as compared with cervical cancer patients without distant metastasis (95% CI 1.91, 86.13; p = 0.009). For with every 1-mm increment in tumour size, cervical cancer patients had a 1.08 odds chance of having *ADAM9*-positive expression (95% CI 1.02, 1.13; p = 0.004).

## Discussion

In Malaysia, the majority of cervical cancer cases occur in women aged 30–59 years old (Manan et al., 2015). In the current study, the mean (SD) age of the patients diagnosed with cervical cancer was 53.89 (10.83) years old. In our study, 76 (80.0%) of the cervical cancer patients were Malay, 15 (15.8%) were Chinese, 1 (1.1%) was Indian, and 3 (3.2%) were other races. According to the National Cancer Registry in 2006, cervical cancer in Malaysia was higher among Malays and Chinese (47.7% and 42% respectively) and lower among Indians (10.3%) (Manan et al., 2015). The findings in the present study on the incidence of cervical cancer among the different races can most probably be explained by the study location (Kelantan, Malaysia), where Malays account for the majority of the population, with Chinese, Indians and other races making up a minority. In the present study, the mean age (SD) at the time of the first pregnancy among the cervical cancer patients in HUSM was 21.87 (2.49) years.

According to the WHO, 85% of all cervical cancers are squamous cell carcinomas, followed by adenocarcinomas (10–12%) and other types of malignancy (3–5%). In our study, although the percentage of squamous cell carcinomas (70.5%) was lower than that in the WHO report, they accounted for this majority of cervical cancer cases. The most likely explanation for these findings is that HUSM is a tertiary (referral) centre in Kelantan. Thus, all the patients diagnosed or treated in HSUM were referred from other primary or secondary centres.

In the present study, 72 (75.8%) samples were positive for *ADAM9* expression. The remainder of the samples were negative for *ADAM9* expression. These findings are in contrast to those presented earlier (Zubel et al., 2009) in which ADAM9 protein expression was found in 93% of cervical cancer patients. The discord between the findings of the two studies is most probably due to methodological differences (i.e. differences in the antibodies, incubation times and target retrieval solutions used and ADAM protein studied).

In the present study, 52 (72.2%) squamous cell carcinoma, 18 (25.0%) adenocarcinoma and 2 (2.8%) adenosquamous cell carcinoma samples were positive for ADAM9 immunostaining (Table 2). Thus, the prevalence of *ADAM9* expression appears to be high in aggressive types of tumour (i.e. squamous cell carcinomas). Positive ADAM9 immunostaining was also observed in tumours with a mean (SD) size of 40.13 (16.25) mm which indicates that the prevalence of *ADAM9* expression is high in large (aggressive) tumours. Twenty-three (31.9%) cases with lymph node involvement and 39 (54.2%) cases with distant metastasis were positive for *ADAM9* expression. The high prevalence of *ADAM9* expression in patients with lymph node involvement and in patients with distant metastases showed that *ADAM9* expression increased in aggressive tumours. These findings are consistent with those of a previous study of renal cell carcinomas (Fritzsche et al., 2008). 

In the study, ADAM9 was highly expressed in high-grade tumours and in tumours with distant metastasis and lymph node involvement. The expression of *ADAM9* in the present study is consistent with that reported in squamous cell carcinomas in the study by Zubel et al., (2009). As shown by the results of the present study, *ADAM9* was expressed in both adenocarcinomas and adenosquamous cell carcinomas. Given the expression of *ADAM9* in various types of cervical carcinomas, the findings presented herein could serve as a stepping stone for further studies on the relationship between *ADAM9* expression and tumorigenesis.

The results revealed no association between *ADAM9* expression and the following patient-related parameters: age, race, smoking status or co-morbid diseases. A previous study also found no association between ADAM 9 expression and the age of the patient (Fritzsche et al., 2008).

*Limitation and recommendation*

A limitation of this study was the lack of an analysis of all specimens from cervical cancer patients diagnosed or treated in HSUM that were suitable for inclusion in the research due to missing or damaged paraffin blocks. Although information on relevant patients was found by database screening, these patients could not be included in the study due to the missing/damaged paraffin blocks. However, the number of specimens included in the sample was adequate based on the calculated sample size and was within the 80% power of the study.

## Ethical approval

Ethical clearance was obtained from Human Research Ethics Committee (HREC) Universiti Sains Malaysia (Ref: USMKK/PPP/JEPeM [243.3.(15)]. All stages of this study adhere to the Declaration of Helsinki’s principles.

## Funding Statement 

This research was funded by the Short-Term Grant, Universiti Sains Malaysia.

## Conflict of Interest

There was no conflict of intrest.
